# Comparison of Fracture Resistance of Endodontically Treated Teeth With Traditional Endodontic Access Cavity, Conservative Endodontic Access Cavity, Truss Endodontic Access Cavity, and Ninja Endodontic Access Cavity Designs: An In Vitro Study

**DOI:** 10.7759/cureus.28090

**Published:** 2022-08-17

**Authors:** Prasad Patil, Pooja Newase, Swapnil Pawar, Hasmukh Gosai, Dharmendra Shah, Sameer M Parhad

**Affiliations:** 1 Department of Endodontics, Al Ameen Dental College, Bijapur, IND; 2 Department of Conservative Dentistry and Endodontics, Dr. D.Y. (Dnyandeo Yashwantra) Patil Dental College and Hospital, Pune, IND; 3 Department of Endodontics, Yogita Dental College, Khed, IND; 4 Department of Conservative Dentistry and Endodontics, Siddhpur Dental College and Hospital, Patan, IND; 5 Department of Prosthodontics, Siddhpur Dental College and Hospital, Patan, IND; 6 Department of Orthodontics and Dentofacial Orthopaedics, Dr. Rajesh Ramdasji Kambe Dental College and Hospital, Akola, IND

**Keywords:** ninja access cavity, truss endodontic cavity, conservative endodontic cavity, fracture resistance, endodontic access cavity

## Abstract

Introduction: Endodontic access cavity preparation plays a vital role as preservation of enamel structure is of utmost importance for a tooth's strength to be maintained. As teeth become fragile after a root canal therapy, this study was designed to compare in vitro the fracture resistance of root-filled and restored teeth with traditional endodontic access cavity, conservative endodontic access cavity (CEC), ninja endodontic access cavity (NEC), and truss endodontic access cavity (TEC).

Materials and methods:* *Control (intact teeth) and traditional endodontic access cavity as well as CEC, NEC, and TEC groups were each given a new human mandibular molar that was freshly removed. Cone beam computed tomography (CBCT) scans of the cone beam showed the values of CEC, NEC, and TEC. After that the teeth were endodontically treated and repaired. To test the specimens, universal testing equipment was used. In order to avoid tooth breakage, the maximum load was determined. Statistical analysis was used in the form of Kolmogorov-Smirnov and Levene tests, which were used to examine data for typical dispersion and consistency in change.

Results:* *Intact teeth showed the highest resistance to fracture compared with other groups. TEC showed significantly higher resistance to fracture compared to the CEC design.

Conclusions: It is possible, within the restrictions of this research, to infer that the TEC design enhanced tooth fracture strength in comparison with the CEC design.

## Introduction

Access cavity preparation is the most significant development in effective endodontic treatment [1]. Only minimal changes have been made throughout time to the typical endodontic cavity preparation for different kinds of teeth [2]. The tooth's ability to break under helpful stresses may be weakened if the necessary tooth structure is expelled during the cavity preparation procedure [3,4]. Extraction is the most common consequence of cracks in teeth treated with endodontics [5-7]. Excessive endodontic access pit design reduces sound dentin and increases tooth deformability, putting the integrity of endodontically treated teeth at risk of fracture [8-11]. Conservative endodontic access cavity (CEC) design has been considered recently as a means of minimizing tooth structure ejection while preserving some of the chamber roof and pericervical dentin [12,13]. With the use of cone beam computed tomographic imaging (CBCT), this sound dentin protection may be performed [14-15]. Endodontically treated teeth may be able to shatter even stronger as a result [12].

A new, ultra-safe approach has just been presented and is often referred to as "ninja." Using this method, endodontically treated teeth may have a lesser chance of breakage than untreated teeth [16]. In contrast to the comparison between conventional endodontic access cavity and ninja endodontic access cavity (NEC) and support, there have been no studies comparing the CEC preparation and the NEC preparation until now. We set out to determine the fracture strength of teeth that had had endodontic treatment by employing standard endodontic access cavity, CEC, NEC, and truss endodontic access cavity (TEC) preparations.

## Materials and methods

Specimen selection and preparation

The study was conducted at Vasantdada Patil Dental College and Hospital, Sangali, Maharashtra, India. Fifty mandibular molars were chosen for periodontal extraction. The chosen ones had fully developed apices. Exclusion criteria included teeth with caries, prior restorations, and apparent fracture lines or fissures. All of the teeth were X-rayed and those that did not match the inclusion criteria were excluded.

It was important to keep the teeth hydrated during the experiment; thus, they were stored in containers with individual numbers and 0.1% thymol solution at 4^0^C. The teeth were sorted into five groups of 10 teeth each. In order to reduce the impact of tooth size and shape differences, we constructed the following homogeneous groups. Group 1: Control, Group 2: Traditional endodontic access cavity, Group 3: CEC, Group 4: NEC, and Group 5: TEC.

**Figure 1:**
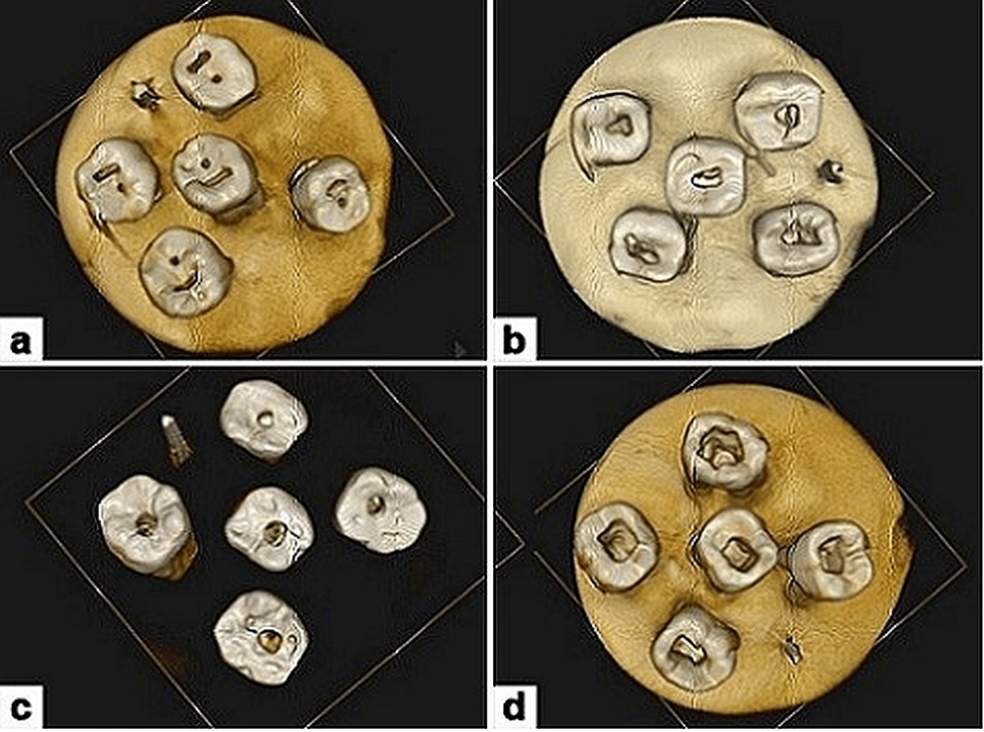
Different types of access cavity preparation a: Truss access cavity preparation; b: Conservative access cavity preparation; c: Ninja access cavity preparation; d: Traditional (conventional) access cavity preparation

CEC, NEC, and TEC teeth were placed in polyvinyl siloxane impression material in contrast to teeth in the control group that had their normal endodontic access cavity preparation. In order to design the access cavity, a CBCT scanner (Kodak 9000 3D; Carestream Health, Inc, Marnela-Vallee, France) was used to capture images of every tooth with a spatial resolution of 200 mm. Traditional endodontic access cavity, CEC, NEC, and TEC outlines are shown in Figure 1.

We used a high-speed handpiece with water cooling to bore the standard endodontic access cavity preparation for all teeth; size 856 diamond burs (Komet Italia SRL, Milan, Italy) were employed. The teeth in the traditional endodontic access cavity group were organized in accordance with the newly disclosed norms of conventional endodontic access cavity preparation. Molars were accessed at the mesial quarter of the central fossa, and cavities extended apically and distally while maintaining part of the chamber roof. Molar teeth were treated similarly to those found in groups of the CEC, although chamber roofs were maintained as much as possible in groups of NEC. Oblique projection toward the central fossa of the root canal orifices on the occlusal plane provided the ninja frame for the entry. Having the endodontic access and the façade cut at 90^o^ or more to the occlusal table, it was possible to minimize all root channel openings, even from diverse visual perspectives.

Size #2 Endo-Access unit (Maillefer Instruments Holding, Ballaigues, Switzerland) was positioned buccolingually along the long central portion of the tooth to get access to a pulp chamber. It was then placed on top of the distal pulpal horn and the chamber was made accessible [17].

**Figure 2:**
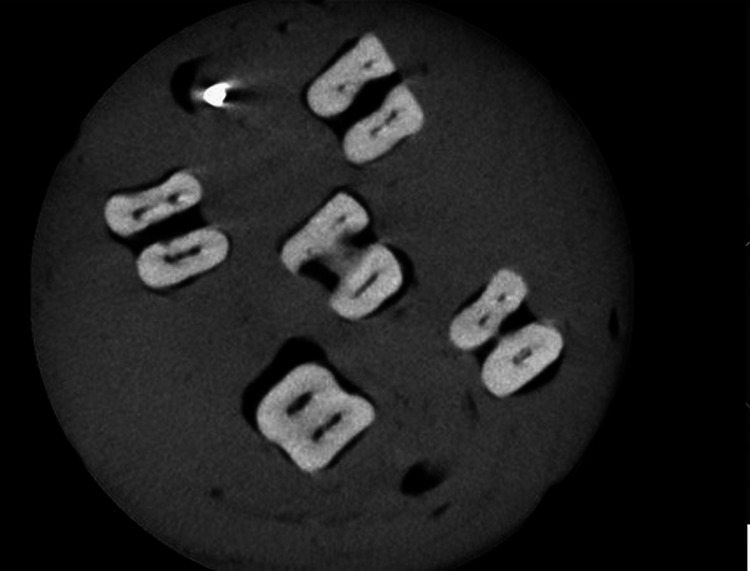
Cone beam computed tomography images of the root canal

This was followed by an examination of all teeth in each group using CBCT imaging as illustrated in Figure 2.

Endodontic treatment

During the cleaning and shaping procedure, protaper rotary files were used to clean and shape the root canals. SX-S2 was used to shape the top 66% of the root canal, while F1-F2 was used to finish the brushing motion. Endo-Prep-RC (Anabond Limited, Chennai, India) and sodium hypochlorite at 5.25% were alternatively used and irrigation was performed. Ultrasonic and manual extractions of the pulpal tissue with 5.25% sodium hypochlorite were used to ensure thorough root canal cleansing. Paper points were used to dry the canal, which was then filled with AH Plus Root Canal Sealer (Maillefer Instruments Holding, Ballaigues, Switzerland), sealant, and gutta-percha (single-cone size 25, 0.06).

Teeth restoration

We used 37% phosphoric acid to clean and etch the access cavity, then we washed it for 30 seconds with a water/air spray and gently dried it to avoid parching. After being applied and allowed to air-dry, the holding expert Prime-Dent light-fix dentin holding gum (Prime Dental Manufacturing (PDM), Inc., Chicago, Illinois, United States) was subjected to exposure for a period of 30 seconds at a light-producing diode. In the end, the composite was used for the restoration of the cavity (3M ESPE; 3M Deutschland GmbH, Neuss, Germany).

Fracture test

At the cemento-enamel junction, 50 teeth's foundations were implanted in acrylic resin blocks. The teeth were loaded at their central fossa. The continuous compressive force at a crosshead speed of 0.5 mm/min was applied using a 6-mm-diameter ball-ended steel compressive head. The loads at which the teeth were fractured, indicated by the software of the load testing machine, were recorded in newtons.

Statistical analysis

Data were initially tested using the Kolmogorov-Smirnov and Levene tests for typical dispersion and homogeneity of changes, respectively. Thus, students were able to get a true picture of their progress via the use of the Student-Newman-Keuls test for correlations.

## Results

Intact teeth showed the highest resistance to fracture compared with other groups. TEC and NEC showed significantly higher resistance to fracture compared to traditional and CEC designs. The TEC and NEC groups had no significant differences in fracture resistance (P >.05) (Table 1).

**Table 1:** Distribution of mean scores of different access preparations

	Mean	Std. Deviation	F	P value
Control	2116.400	1570.6339	2.029	.129
Traditional	702.000	308.6835		
Conservative	944.600	520.4871		
Truss	1805.800	981.2641		
Ninja	1712.200	827.2144		
Total	1456.200	1024.5293		

## Discussion

Too much crown material is removed during endodontic treatment, which may lead to fractures in the root-filled teeth. According to one study, the second leading cause of tooth structural loss was improper preparation of the endodontic access cavity using typical endodontic access cavity methods [18]. A better prognosis for a tooth may be achieved by removing as little of the crown as possible during access cavity preparation. It has been established that the use of CEC, NEC, and TEC designs in endodontically treated teeth reduces the risk of breakage. On teeth that will be treated endodontically, such as those with periodontal disease or those that have had a deliberate root canal procedure, these techniques may be used clinically. According to the authors' statistics, only 8% of the patients they treated in the preceding five years had this particular clinical condition [1].

Only a few studies have looked at the fracture strength of teeth with CEC and NEC access, and no studies have looked at the teeth on their whole. Endodontically treated teeth were put to the test to see how well they held up in a fracture test. The use of completely grown, complete mandibular molars helps to minimize dental structural loss [18,19]. Due to teeth's tendency to fracture under eccentric stresses [20], the 30^o^ inclination angle was used since the failure threshold for eccentric fractures is lower than the axial fracture loads of past studies. Access cavities were reconstructed utilizing bonded resin composite in order to imitate clinical procedures and facilitate loading testing. When endodontic access cavities are healed, the fracture strength of teeth may be recovered to up to 72% of that of undamaged teeth [21].

For consistency's sake, all specimen preparation procedures were carried out by the same experienced hand. When it came to fracture strength, the traditional endodontic access cavity group was the weakest in this research. Prior research found similar findings when teeth were evaluated without any restorations, which is not typical in the dental field. Dentin protection achieved by reducing cavity size has been shown to increase tooth break strength in the continuing study, which is consistent with previous findings [22,23]. No matter what kind of material was used in tooth buildup, there was no difference in fracture strength between the NEC and TEC in any of the studied teeth. The NEC and TEC cavity may have a higher break strength than the standard endodontic access cavity, but they also increase the risk of procedural mistakes due to the absence of canal instrumentation. All pulp tissue, debris, and necrotic materials should be removed from the optimal access pit. As a result, a smaller access cavity increases the risk of bacterial contamination and the possibility of missing some root canal orifices [24]. The fracture strength of teeth with CEC access and conventional endodontic access cavity was further enhanced by NEC and TEC in this research.

The limitations of the study are the sample size and the in vitro studies. In vivo studies are more relevant. However, in them, more clinical exams are needed to determine the appropriateness of the equipment, difficulties during endodontic procedures, and the long-term prognosis of endodontically treated mandibular molars with CEC, NEC, and TEC.

## Conclusions

The endodontic access cavity helps to extirpate pulp tissue and also maintain tooth integrity before the restoration. Tooth structure is of the utmost importance when we talk about strength after the root canal treatment. If the cavity preparation is very small and there is minimal loss of tooth structure, the subsequent teeth will have a greater capacity for withstanding the masticatory forces since they will have more strength. The truss endodontic cavity increases the fracture strength of teeth compared with the conservative access cavity. The fracture strength of teeth is because of dentin preservation obtained by cavity size reduction. But no difference in the fracture strength was observed among the NEC and TEC in all tested teeth.
